# Anaplastic Thyroid Cancer in Sicily: The Role of Environmental Characteristics

**DOI:** 10.3389/fendo.2017.00277

**Published:** 2017-10-20

**Authors:** Martina Tavarelli, Pasqualino Malandrino, Paolo Vigneri, Pierina Richiusa, Adele Maniglia, Maria A. Violi, Giulia Sapuppo, Veronica Vella, Gabriella Dardanoni, Riccardo Vigneri, Gabriella Pellegriti

**Affiliations:** ^1^Endocrinology, Department of Clinical and Experimental Medicine, University of Catania, Garibaldi-Nesima Medical Center, Catania, Italy; ^2^Medical Oncology, Department of Clinical Experimental Medicine, University of Catania, Policlinic Hospital, Catania, Italy; ^3^Endocrinology, Di.Bi.M.I.S., University of Palermo, Palermo, Italy; ^4^Department of Clinical and Experimental Medicine, University of Messina, Messina, Italy; ^5^Motor Sciences, School of Human and Social Sciences, “Kore” University, Enna, Italy; ^6^Osservatorio Epidemiologico Regionale, Assessorato Salute Regione Siciliana, Palermo, Italy; ^7^Institute of Biostructure and Bioimaging, CNR, Catania, Italy

**Keywords:** thyroid cancer, anaplastic thyroid cancer, thyroid cancer epidemiology, Thyroid Cancer Registry, thyroid cancer and volcanic environment

## Abstract

**Background:**

Anaplastic thyroid cancer (ATC) is a rare but extremely aggressive cancer of the thyroid, contributing up to 30–40% of thyroid cancer-specific mortality. We analyzed ATC characteristics and survival rates in Sicily to evaluate the possible influence of environmental factors. With this aim, data regarding ATC incidences in urban/rural and industrial, iodine-deficient, and volcanic vs control areas were compared in Sicily as well as ATC data from Sicily and USA.

**Methods:**

Using the Sicilian Register of Thyroid Cancer (SRTC) database incidence, age, gender, tumor size and histotype, extrathyroidal extension, stage, and coexistence with pre-existing differentiated thyroid cancer (DTC) were evaluated in different areas of Sicily and also compared with Surveillance Epidemiology and End Results data in USA.

**Results:**

Forty-three ATCs were identified in Sicily in the period 2002–2009. In our series only age <70 years at diagnosis (*p* = 0.01), coexistence with DTC (*p* = 0.027) and tumor size ≤6 cm (*p* = 0.012) were significant factors for increased survival at univariate analysis (only age at multivariate analysis). No difference in ATC incidence was found in urban vs rural areas and in iodine-deficient and industrial vs control areas. By contrast, in the volcanic area of Sicily, where DTC incidence is doubled relative to the rest of the island, also ATC incidence was increased. ATC data in Sicily were similar to those reported in the same period in the USA where overall survival rate at 6 and 12 months, however, was smaller.

**Conclusion:**

The similar ATC data observed in Sicily and USA (having different genetic background and lifestyle) and the increased ATC incidence in the volcanic area of Sicily paralleling the increased incidence of papillary thyroid cancer are compatible with the possibility that casual additional mutations, more frequent in a background of increased cell replication like DCT, are the major causes of ATC rather than genetic background and/or direct environmental influences.

## Introduction

Thyroid cancer is the most common endocrine tumor, making up 3.8% of all new cancers in the USA (1), and in the last three decades its incidence has continuously increased all over the world (2). Most thyroid cancers are well differentiated and have an indolent evolution with a low mortality rate (approximately 0.5%), which has been relatively stable over the last decades but has been increasing in the recent years (3).

A specific thyroid cancer histotype, the anaplastic thyroid cancer (ATC) is the major contributor to thyroid cancer-related mortality. In spite of its rarity (1–2% of all thyroid cancers) ATC contributes to 15–50% to the annual mortality of thyroid cancer (4). ATC in fact is one of the most aggressive and deadly human cancers, with a disease-specific mortality close to 100% (5). ATC originates from the thyroid follicular epithelium but does not show any of the biological features typical of the differentiated follicular cells [response to TSH, iodine uptake, and thyroglobulin (Tg) synthesis]. ATC, therefore, is also different from the poorly differentiated thyroid cancers (DTCs) which maintain some of the immunohistochemical markers of the epithelial thyroid cell, such as Tg and thyroid transcription factor 1 (TTF1) (6, 7).

According to Surveillance Epidemiology and End Result (SEER), the ATC incidence is 1–2 cases/million inhabitants/year, and the trend, at variance with well-DTC, shows a progressive decrease (8). A dramatic reduction of ATC has been observed in the last three decades of the last century in Germany and Ireland and is attributed to the increase of dietary iodine and better treatment of DTC (9, 10). Nowadays ATCs represent 1.7% of all thyroid cancers in the United States (11). Based on either tumor registries or single center experience, the ATC frequency among all thyroid cancers is highly variable in different countries, being 1.3% in Australia, 1.9% in Luxembourg, 2.0% in Austria, 2.1% in Italy, 3.1% in Japan and Jordan, 4.2% in New Zealand, 4.7% in India, 7.5% in Israel, 7.9% in Netherlands, and 9.8% in Ireland (12).

Because of its aggressive behavior, ATC is classified as T4 and Stage IV cancer (IV A/B/C) by the latest American Joint Committee on Cancer Staging Manual (13). The median survival of ATC patients is 3–5 months after diagnosis with a survival rate of 20% at 1 year and less than 5% at 5 years (4).

Anaplastic thyroid cancer patients are generally older than patients with DTC with a peak of incidence in the sixth to seventh decade of life and less than 10% of cases younger than 50 years. The female to male ratio is lower than in DTC, between 2:1 and 3:1 (4, 14). ATC often occurs in patients with a pre-existing goiter (up to 80% of cases) or a DTC. More than 90% of ATC tumors are locally invasive (15, 16) and present a rapidly growing fixed neck mass, invading surrounding tissues with neck lymph node involvement (17). Therefore, local symptoms, including neck pain, tenderness, and compression (or invasion) of the upper aerodigestive tract, with dyspnea, dysphagia, hoarseness, and cough usually prevail on systemic symptoms (anorexia, weight loss, fatigue, shortness of breath, and fever of unknown origin) that appear only when the disease is more advanced (4). Fifty percent of patients present with distant metastases already at diagnosis: lung (80%), bone (6–16%), and brain (5–13%) are the most common metastasis sites (15).

Due to these characteristics at presentation ATC should be considered a systemic disease that requires multimodal therapy involving reductive surgical resection in addition to external beam radiation therapy and chemotherapy (doxorubicin and paclitaxel), either concurrently or sequentially (4). Hopefully, in the near future, a better understanding of the genomic and transcriptomic hallmarks of ATC (18) will allow for the development of new therapeutic strategies based on molecular approaches with single (19, 20) or multiple molecular target approaches (21–25).

The aim of this study was to analyze demographic and clinical characteristics of ATC relative to different environmental influences in individuals with a similar genetic background and lifestyle. ATC incidence was examined in rural vs urban areas of Sicily as well as in iodine-deficient areas and in industrialized areas and data compared to values in the rest of the island. Moreover, ATC was separately evaluated in the area of increased incidence of thyroid cancer identified around the Mt. Etna volcano (26). In that volcanic area, thyroid cancer incidence is doubled relative to the rest of Sicily and the increase is exclusively due to the papillary histotype (26). This increase of thyroid cancer in the volcanic area is associated with important non-anthropogenic environmental pollution. Analyzing 27 trace elements in drinking water, lichens, and in urines of residents in the volcanic area and in adjacent non-volcanic Sicilian areas, we found that values of many metals were significantly increased in water and atmosphere of the volcanic area with consequent biocontamination of the resident population (27, 28). In particular, average values of palladium (×9), vanadium (×8), manganese (×3), mercury (×2.6), tungsten (×2.4), and cadmium (×2.1) were the major biocontaminants identified (27–29).

Moreover, to evaluate the possible influence of the different genetic background, ATC data in Sicily were compared with those available from the SEER program in the USA. Survival rates and prognostic factors were also calculated. All these comparisons were aimed at evaluating the possible role of environmental characteristics on the incidence of ATC in populations living in adjacent areas and having a similar genetic background and lifestyle (Sicily) and also in two countries (Sicily and USA) with very different genetic background and lifestyle.

## Patients and Methods

### Data Source

#### The Sicilian Regional Register for Thyroid Cancers (SRRTC)

The Sicilian Regional Register for Thyroid Cancers (SRRTC) was established in Sicily in January 2002 and is based on a double independent identification system: (a) active search in all Sicilian pathology centers for histological diagnosis and (b) systemic analysis of the regional data-base of the hospital discharge records (SDO) and death certificates.

The files of the National Health Service hospital’s discharge forms were provided by the Sicilian Epidemiological Observatory classified according to the *International Classification of Diseases, Ninth Revision, Clinical Modification*, and including the diagnoses of thyroid cancer (ICD-9-CM code 193), thyroidectomy (ICD-9-CM code 064) and acquired hypothyroidism (ICD-9-CM code 2440). Only patients with a histopathology diagnosis of thyroid cancer according to the International *Classification of Diseases for Oncology* (ICD-O code C73.9) were included. Data were collected in a computerized folder that includes demographic (age, gender, residence) and clinical data (type of surgery, histological type, tumor size, extrathyroidal extension, disease staging). All diagnoses of ATC from January 2002 to December 2009 were included and cases were staged according to the TNM system (seventh edition).

### SEER Data

The SEER program collects data on all malignancies diagnosed through histology in all SEER eligible cancer registers. We obtained data on ATC (ICD-O-3 8012, 8020, 8021, 8030-32) from the SEER 18 registries (released April 2016, based on the November 2015 submission) through the SEER*Stat software version 8.3.2. Only ATC diagnosed in the same period of the SRRTC studied series (2002–2009) were selected.

### Geographic and Environment Characteristics of Sicily

Sicily is a Mediterranean island with a homogenous and stable population of 5,013,000 inhabitants in 2005 (http://demo.istat.it/pop2005/index.html).

The geographic and environmental characteristics of Sicily are different in different areas. The population is distributed in nine provinces. Nearly 25% of the Sicilian population lives in rural areas (defined as having a population density of ≤150 inhabitants per square kilometer) and approximately 8% in extensively industrialized areas (mostly chemical and mechanical installations) (26). Nearly 5% of Sicilians live in areas of mild-to-moderate iodine deficiency, scattered over the island, but mostly in the central highlands. The iodine-deficient population was determined by more than 30 years of continuous studies on endemic goiter, including goiter surveys in schoolchildren and measurements of urinary iodine and radioiodine thyroid uptake in adults (30–32). Finally, nearly 20% of the Sicilian population live in the volcanic area of Mt. Etna, the largest and most active volcano in Europe. This area includes most of the province of Catania (approximately 80% of residents) and part of the province of Messina (approximately 25% of residents).

### Statistical Analysis

Mean ± SD is provided for continuous variables, whereas categorical parameters are shown as number and percentage. Quantitative and qualitative data were compared using the *t*-test and the chi-square test, respectively. To calculate the age-standardized incidence rate of ATC on the world population (ASRw), data on the numbers of newly diagnosed ATC every year and population size for SRRTC were obtained from the Italian Institute for Statistics while for the USA data from SEER registry were analyzed.

The comparisons of the incidence of thyroid cancer in different geographical areas were performed using Poisson regression analysis, including age and gender in the model. Survival rates were calculated according to the Kaplan–Meier method. Statistical analyses were performed using commercially available software (Stata version 13.1; StataCorp, College Station, TX, USA) and a *p*-value of less than 0.05 was considered statistically significant.

### Ethical Approval

Data on patients with thyroid cancer were collected following the privacy data directive of the European Union (26). The use of SRRTC data has been authorized by the Sicilian Regional Epidemiological Observatory. Ethical approval is not required and informed consent is waived since this is a retrospective study and individual data are collected anonymously.

## Results

### ATC Incidence and Characteristics in Sicily

Forty-three ATCs were identified among the 5,328 thyroid cancers diagnosed in Sicily between 2002 and 2009 (0.8% of all thyroid cancers in the same period) with an overall age-standardized incidence rate (ASRw) of 0.05/10^5^ inhabitants/year, identical in males and females.

In our series, ATC patients were more frequently females (25 cases vs 18 in males) with a female to male ratio of 1.4/1, much smaller than the overall thyroid cancer F/M ratio in Sicily (4.3/1) (33).

The mean age at diagnosis was 71.6 years (range 51.4–95.3) and was lower in males (69.7 ± 6.2 years) than in females (73.0 ± 11.4 years), but the age difference between the two genders was not statistically significant (*p* = 0.28). No ATC patient was younger than 50 years at presentation and in most cases (25/43, 58.1%) the age at diagnosis was ≥70 years (Table 1); the ASRw was 0.5/10^5^ inhabitants/year among individuals aged ≥70 and was nearly 10 times lower in patients aged <70 years (0.05/10^5^ inhabitants aged 50–70 years) (*p* < 0.001).

**Table 1:** Anaplastic thyroid cancer (ATC) patients and tumor characteristics in Sicily and in USA (2002–2009).

	Sicily	USA	*p*
**N. cases**	43	683	
**ASRw**	0.05	0.06	N.S.
**Mean age (± SD)**	71.6 (±9.7)	70.3 (±9.7)	0.5
F (N. 25)	73.0 (±11.4)		
M (N. 18)	69.7 (±6.2)		
**F/M ratio**	1.4/1.0	1.6/1.0	0.62
**Tumor size^a^**	5.7 (±2.4)	6.3 (±2.9)	0.27
≤6 cm	23/33 (69.7%)		
>6 cm	10/33 (30.3%)		
**Extrathyroid invasionb**			
Present	27 (73%)		
Absent	10 (27%)		
Coexistence with DTC	20 (46.5%)		
**Staging (TNM)**			
IVA	16 (37.2%)	69 (10.1%)	<0.001
IVB	26 (60.5%)	571 (83.6%)	
IVC	1 (2.3%)	43 (6.3%)	
**Survival**			
Median (months)	6.8	5.0	0.28
Survival rate 6 months	51.2%	38.0%	0.09
1 year	37.2%	25.0%	0.08

*^a^Available in 33 patients*.

*^b^Available in 37/43 patients*.

*p: USA vs Sicily*.

*N.S, non-significant*.

The median tumor size at presentation was not available or not measurable in 10 patients. It was 5.7 cm (±2.4) in the remaining 33 patients with no significant difference between gender (*F* = 5.5 ± 2.7 cm; M = 6.0 ± 1.9 cm; *p* = 0.56). In younger patients (<70 years), the median tumor size was significantly smaller than that in patients ≥70 years (4.3 vs 6.0 cm, respectively) (*p* = 0.02).

Extrathyroid extension at presentation was evaluated in 37/43 patients and it was present in 27/37 cases (73%), and was more frequent in females (17/22 cases or 77.3%) than in males (10/15 cases or 66.7%). This difference is not significant (*p* = 0.47). Extrathyroid extension was similar in older (≥70 years) patients (17/24 or 70.8%) and younger patients (<70 years, 10/13 or 76.9%, *p* = 0.69).

The ATC developed in normal or goitrous non-malignant thyroid in 23 patients whereas it coexisted with differentiated thyroid carcinoma in 20 patients (46.5%): 11 papillary classic variant, 1 tall cell and 1 papillary follicular variants, 1 poorly differentiated, and 6 follicular cancers.

The histo-pathological characteristics of ATC were available in 19 cases: 6 giant cell, 4 spindle cell 3 squamoid cell and 6 mixed variants (spindle/giant, squamoid/giant, spindle/squamoid) were identified.

After diagnosis, patients were staged according to the TNM: 16 patients were stage IVA, 26 IVB, and 1 was stage IVC. According to gender, stage IVA was more frequent in men (55.6%) than in women (24.0%) although the difference did not reach statistical significance (*p* = 0.08).

No standardized treatment was used in this cohort since patients were followed by different multidisciplinary teams with the coordination of either endocrinology or surgery or oncology specialists. Detailed information on surgery was available in 41/43 patients. Most had total thyroidectomy (29/41) with a subsequent enlarged lymph node dissection in five cases. Partial thyroidectomy (mass debulking) was carried out in 10 patients, while only a diagnostic core biopsy was carried out only in 2 patients because neck surgery was not suggested due to the locally advanced infiltrative disease or the presence of distant metastases.

### Risk Factors and Survival Analysis

In the 43 ATC patients diagnosed in Sicily, the unadjusted median survival was 6.8 months (95% C.I. 4.6–16.8), longer in males (16.8 months) than in females (4.8 months) (*p* = 0.19) and in younger patients (44.5 months in patients <70 years vs 4.6 in patients ≥70 years) (*p* < 0.001). The survival was slightly but not significantly longer when a DTC coexisted (7.2 months vs 4.6 months in patients without a DTC, *p* = 0.18). Considering the histotype, survival was longer in patients with squamoid ATC (8.8 months) vs 4.4 months in patients with a mixed histological pattern, 2.1 months in giant cell ATC and 1.3 months in spindle cell ATC. The overall survival rate was 51.2% at 6 months and 37.2% (16/43) at 1 year.

There were 14/43 (32.6%) long-term ATC survivors (surviving >2 years) and they were characterized by a significantly younger mean age (*p* < 0.006, Table 2) with a significantly smaller percentage of patients aged more than 70 years. Longer survivors were more frequently associated also with the male gender, a smaller tumor size and a more frequent coexistence of a DTC. All these differences between long-term ATC survivors and the other ATC patients, however, were not statistically significant (Table 2). The differences regarding the absence of extrathyroid extension and stage IVA were nearly significant (*p* = 0.06), being both more frequent in long-term survivors (Table 2).

**Table 2:** Comparison between long-term anaplastic thyroid cancer (ATC) survivors and other ATC patients in Sicily.

	Long-term ATC survivors^a^ (*n* = 14)	Other ATC patients (*n* = 29)	*p*
Mean age (years)	67.0 (±7.0)	73.8 (±10.0)	0.03
Age >70 years	4 (28.6%)	21 (72.4%)	0.006
F/M ratio	0.75/1	1.9/1	0.16
Coexistence with DTC	8/14 (61.5%)	12/29 (42.9%)	0.27
Tumor size (cm, M ± SD)	4.9 (±1.6)	6.0 (±2.5)	0.23
Extrathyroid invasion^b^	5/10 (50%)	22/27 (81.5%)	0.06
**Stage**			
IV A (*n* = 16)	8/14 (57.1%)	8/29 (27.6%)	0.06
IV B + IV C (*n* = 27)	6/14 (42.9%)	21/29 (72.4%)	

*^a^Surviving beyond 2 years*.

*^b^Available only in the 37/43 patients*.

In the univariate analysis factors associated with lower risk of ATC-related death were young age, tumor size ≤6 cm and coexistence with DTC, whereas the male gender, extrathyroid invasion and non-advanced stage were not significant risk factors (Table 3). Only younger age was a significant factor for better outcome at the multivariate analysis.

**Table 3:** Univariate and multivariate analysis for the risk of anaplastic thyroid cancer (ATC)-related death.

	Univariate Analysis		Multivariate analysis	
Parameter	HR (95%CI)	*p*	HR (95%CI)	*p*
Male gender	0.64 (0.32–1.27)	0.20		
Age at diagnosis	1.08 (1.03–1.12)	0.001	1.06 (1.00–1.12)	0.05
≥70 years	3.64 (1.70–7.80)	0.001	1.13 (0.94–1.37)	0.20
Tumor size	1.23 (1.01–1.48)	0.036		
>6 cm	2.85 (1.26–6.44)	0.012		
Extrathyroid invasion	1.91 (0.81–4.52)	0.14		
Coexistence with differentiated thyroid cancer (DTC)	0.45 (0.22–0.91)	0.027	0.70 (0.29–1.67)	0.42
Stage IVA (*n* = 16)	1.0			
Stage IVB + IVC (*n* = 27)	2.01 (0.97–4.16)	0.059		

*Data at univariate analysis indicate that the risk of ATC-related death increases +8% for the increase of each year of age at diagnosis and +23% for each cm of cancer size increase while it is reduced by 55% when coexisting DTC*.

### Environmental Influence on the ATC Distribution in Sicily

Anaplastic thyroid cancer occurrence was not evenly distributed on the island of Sicily in spite of similar genetic and lifestyle backgrounds and similar access to the National Health Service (26).

Because of the limited number of cases, a statistical analysis of ATC incidence for each of the nine provinces of Sicily was not possible. We first compared the ATC incidence in industrial vs non-industrial areas of Sicily and between residents of rural vs urban areas. No difference in ATC incidence was found according to these environmental characteristics (*p* = 0.50 and *p* = 0.52, respectively). In the areas of mild–moderate iodine deficiency (all rural) and involving only 4–5% of Sicilian inhabitants, no case of ATC was observed in the years between 2002 and 2009.

**Figure 1:**
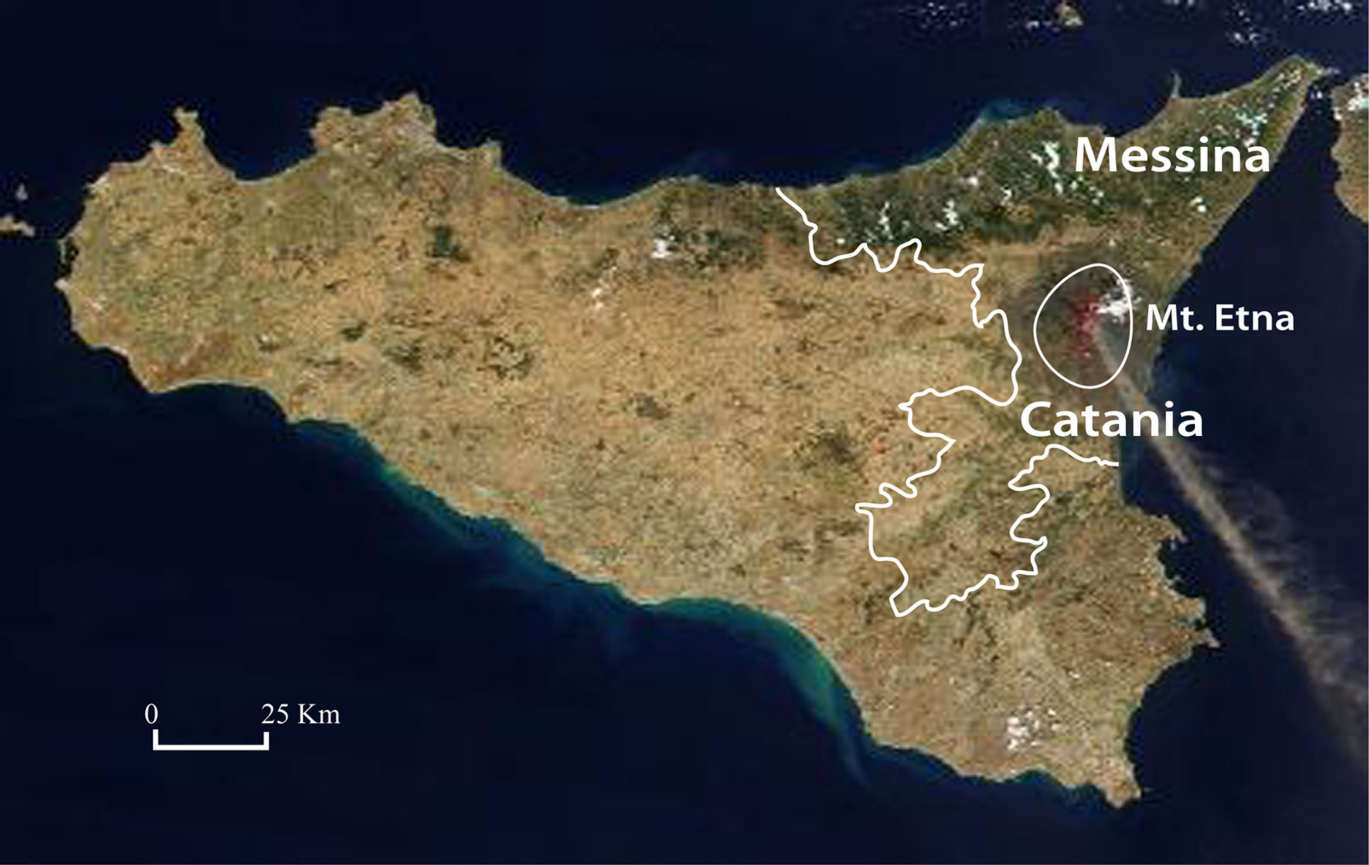
Map of Sicily. The two provinces (Catania and Messina) closer to the MT. Etna volcano are indicated.

When considering separately the province of Catania, where Mt. Etna is located and total thyroid cancer incidence is doubled with a highly significant difference in comparison to the rest of Sicily (ASRw = 31.7, in women and 6.4 in men in the volcanic area vs 14.1 in women and 3.0 in men in the rest of Sicily) (*p* < 0.001), the ATC ASRw was higher in Catania (0.07/10^5^ inhabitants/year) than in the other provinces (ASRw = 0.05) but the difference between the two areas was not statistically significant (*p* = 0.19). However, when the two provinces closest to Mt. Etna were considered together (Catania and Messina, Figure 1) the ATC ASRw was significantly higher (0.07/10^5^ inhabitants/year) in this area than in the rest of Sicily (0.04/10^5^ inhabitants, *p* = 0.048) (Table 4).

**Table 4:** ATC incidence in Sicily and USA.

Geographic area	N. residents (× 1000)	N. cases (2002–2009)	Incidence (ASRw)	*p*
Catania province	1,072	12	0.07	0.19
Rest of Sicily	3,941	31	0.05	
Messina province	658	9	0.07	0.23
Rest of Sicily	4,355	34	0.05	
Catania + Messina provinces	1,730	21	0.07	0.048
Rest of Sicily	3,283	22	0.04	
All Sicily	5,013	43	0.05	0.08
USA	295,520	683	0.06	

### Comparison of ATC in Sicily and USA

Comparing the epidemiological data of ATCs that occurred in Sicily in the period between 2002 and 2009 to those observed in the same period in the USA, no significant difference in the overall incidence was found (*p* = 0.08). Also the mean age at diagnosis and the female to male ratio were similar in the two series.

**Figure 2:**
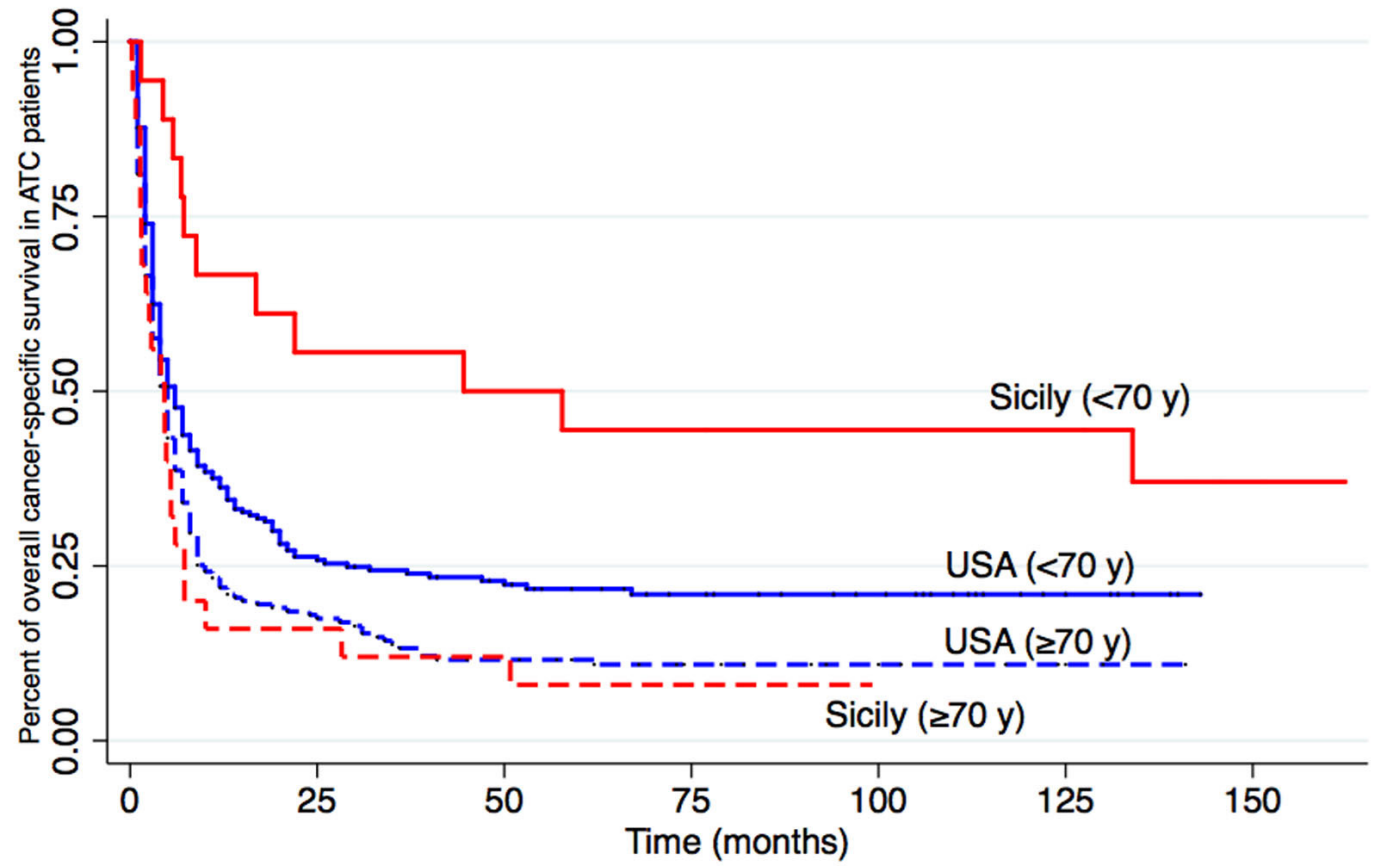
Overall cancer-specific survival. Kaplan–Meier causes for cancer-related mortality in Sicily (The Sicilian Regional Register for Thyroid Cancers, red) and USA (Surveillance Epidemiology and End Results, blue). Anaplastic thyroid cancer patients are subdivided according to age (<70 or ≥70 years) at diagnosis. The log-rank *p*-value is significant (0.02) for the difference in younger patients (<70 years) but not older ones (0.28).

The median tumor size at diagnosis was slightly smaller in Sicily (5.7 vs 6.3). Differences, however, were not statistically significant (*p* = 0.27). But when only patients younger than 70 years were considered, the difference in average ATC size between Sicily (5 cm) and USA (6.2 cm) became nearly statistically significant (*p* = 0.06). In Sicily, a significantly higher prevalence of ATCs in stage IVA was observed (37.2 vs 10.1% in USA, *p* < 0.001, Table 1) and this may have influenced survival. The survival rate was, in fact, better in Sicily (Figure 2) where the median survival was longer (6.8 vs 5.0 months in USA) and also survival rates at 6 months (51.2 vs 38.0%) and at 1 year (37.2 vs 25.0%) were higher (Table 1).

## Discussion

Our study on ATC in Sicily confirms that this cancer is a rare but extremely aggressive malignancy. In comparison with DTC, ATC in Sicily occurs at a more advanced age (mean age 71.6 vs 49.0 years for DTC) (33) and with a much lower female to male ratio (ATC = 1.4 vs 4.3 for DTC). Age, therefore, is a major risk factor and also significantly affects the prognosis: being less than 70 years old significantly improves survival at both univariate and multivariate analysis (Table 3). Furthermore, gender effect is different between ATC and DTC because the worse evolution of the thyroid cancer in males, which is typical of DTC (34–36), is not present in ATC (37–39). In our small series, the younger age and more prevalent IVA stage may have influenced the better survival in male patients relative to females.

In our ATC patients, the coexistence with DTC is associated with a better survival rate at the univariate analysis (*p* = 0.03, Table 3). Although these data are not confirmed at the multivariate analysis, a better prognosis in patients with coexistent DTC is possible because it is influenced by the more precocious ATC diagnosis, when only one or a few ATC foci are incidentally diagnosed at the pathological examination after total thyroidectomy for a DTC. Early diagnosed intrathyroidal ATCs, in fact, are expected to have a better prognosis because ATC foci are completely excised at surgery. When comparing long-term (>2 years) ATC survivors to the other ATC patients (Table 2) age was the only significant factor and the cancer stage was nearly significant.

The possibility of a thyroid hormone role on favoring cell proliferation and ATC *via* activation of the ERK 1/2 intracellular pathway (40) is not supported by our data since no ATC patient had a history of hyperthyroidism or l-thyroxine overreplacement after thyroidectomy.

Environmental factors such as iodine deficiency, living in industrial areas, and in either the rural or urban environment do not influence ATC incidence in Sicily. Moreover, the comparison of ATC data in Sicily with those obtained in the same period (2002–2009) from the SEER series in the USA shows very similar epidemiological characteristics in spite of the different genetic background, lifestyle, and access to medical healthcare. This similarity may suggest that the multistep oncogenic process leading from DTC to ATC (41–43) is driven by chance mutations occurring in an increased replicative situation (DTC) rather than by environmental influences (44). This hypothesis is supported by our observation that ATC incidence is higher in the provinces of Sicily closer to the Mt. Etna volcanic area. In that area, thyroid cancer incidence is doubled in comparison to the rest of Sicily (26, 27, 33, 45). This increase is associated with an important environmental pollution with metals and metalloids and consequent biocontamination of the residents (27). Other volcanic originated pollutants, such as sulfur, thiocyanate, sulfur dioxide, and other volcanic gases, have been hypothesized to influence molecular and cellular mechanisms of thyroid follicular cells favoring cancer (46). Although a cause–effect relationship between this biocontamination and increased cancer incidence is not demonstrated, environmental factors certainly influence the increase of thyroid cancer in volcanic areas (26, 47–49). Since many ATCs occur in long-term pre-existing DTCs by de-differentiation (11), the increased number of ATCs observed in a population having an increased DTC prevalence is in agreement with a minor role of environmental factors and the possibility of ATC incidence related to DTC incidence. This consideration, however, must be considered indirect evidence and does not exclude the possibility that one or more carcinogens in the volcanic areas can directly promote ATC.

One interesting difference between ATC data in Sicily and USA concerns the outcome in terms of overall survival: the percentage of patients surviving at 6 and 12 months after ATC diagnosis is higher in Sicily than in the USA although differences are not statistically significant (*p* = 0.09 and 0.08). This difference becomes significant when only younger (<70 years) patients are considered. Different causes may contribute to the longer survival of ATC patients in Sicily, including the smaller cancer size in Sicilian ATC patients with age <70 years and the less advanced stage at diagnosis (percent of patients with stage IV A 37.2 vs 10.1% in USA, *p* = 0.001). Treatment, of course, is also a factor determining survival (50) and precocious and aggressive multimodality therapy has been demonstrated to be beneficial in the absence of distant metastases (38). Different diagnostic and treatment procedures and different access to the medical system might be responsible for the differences observed between the two countries.

Our study provides no contribution in this regard because treatment of the 43 patients was not performed according to standardized protocols and the ATA guidelines for management of patients with ATC (not yet published when these patients were studied) (11). The heterogeneity of treatment procedures is a major limitation of our study since it may have affected survival. Other limitations, of course, include the small number of cases studied and the limitations common to any retrospective study.

In conclusion, ATC in Sicily was confirmed as a deadly disease with poor long-term survival favored mainly by young age, small tumor size, and a non-advanced stage. The influence of environmental factors in determining ATC incidence is probably minor and indirect: carcinogens might rather be effective at promoting DTC which, in turn, may favor progression to ATC.

## Author Contributions

MT, PM, PR, AM, MV, RV and GP gathered data and drafted the manuscript; MT and PM performed statistical analysis; MT, PM, RV and GP critically reviewed the manuscript. All authors contributed to the conception of the work and approved the final version of the manuscript.

## Conflict of Interest Statement

The authors declare that the research was conducted in the absence of any commercial or financial relationships that could be construed as a potential conflict of interest.
